# Nitrous oxide–induced reproductive risks: Should recreational nitrous oxide users worry?

**DOI:** 10.1177/02698811221077194

**Published:** 2022-05-05

**Authors:** Jan van Amsterdam, Wim van den Brink

**Affiliations:** Department of Psychiatry, Academic Medical Center, University of Amsterdam, Amsterdam, The Netherlands

**Keywords:** Nitrous oxide, laughing gas, recreative drugs, reproduction, congenital anomalies, abortion

## Abstract

**Background:**

Nitrous oxide (N_2_O) is a frequently used anaesthetic. Since the year 2000, recreational use of N_2_O, also known as ‘laughing gas’, became popular as a recreational drug due to its mild *psychedelic* effect. In the 1980s, several reports warned against N_2_O-induced reproductive risks among healthcare personnel, questioning the occupational safety of N_2_O in health care.

**Methods:**

Data about the reproductive risks of N_2_O were collected from literature.

**Results:**

Particularly in the past, professionals working in dental and midwifery practices, operating theatres and ambulance transport were exposed to high levels of N_2_O. Adverse reproduction effects included congenital anomalies, spontaneous abortion and reduced fertility rates in females. Following occupational measures, like maximal exposure limits for ambient N_2_O, this occupational risk was considerably reduced. Recreational users of N_2_O, however, voluntarily and repeatedly expose themselves to (very) high doses of N_2_O. As such, they exceed the health exposure limits some hundred times, but they are fully unaware of the related reproductive risks.

**Conclusion:**

We advocate to increase the awareness in recreational N2O-users about its potential reproductive risks, especially in heavy users, pregnant users or those who intend to become pregnant.

## Introduction

Nitrous oxide (N_2_O; ‘laughing gas’) is a widely used anaesthetic. For over 150 years, N_2_O is used as an anaesthetic and anxiolytic in operating theatres, ambulance transport, dentistry and during labour and delivery. N_2_O has been advocated as a safe standard technique for dentistry and is particularly suitable to treat paediatric patients who are terrified of the dentist (Holroyd and Roberts, 2000; National Institute for Health and Clinical Excellence (NICE), 2010). In the United States, 87–89% of all paediatric dentists have used N_2_O (Mathers, 2018).

In addition to clinical use, recreational N_2_O use has been known since the late 19th century but recently a serious increase in recreational use emerged in many countries (Kaar et al., 2016; van Amsterdam et al., 2022). In the United Kingdom, N_2_O was second only to cannabis in drug use among those aged 16–24 in 2019 (April 2019–March 2020) (ONS, 2020). Recreational N_2_O use became particularly popular in youngsters and adolescents because it is widely available, cheap, legal and most users are convinced that N_2_O is a safe drug (Nabben et al., 2017). For instance, the majority (77%) of N_2_O users in the Netherlands was unaware of the drug’s harmful effects (Kaar et al., 2016). The use of hundreds of bulbs daily for prolonged periods of time and continuous filling and inhaling N_2_O balloons using large 2 kg N_2_O-tanks is emerging (van Riel et al., 2022), though fortunately, this is still an exceptional practice among recreational N_2_O users. These new practices are of concern, because repeated exposure to high doses of N_2_O for a prolonged time is known to induce vitamin B_12_ deficiency and related neurological damage, such as (irreversible) neuropathy and paralysis (for references, see van Amsterdam et al., 2022).

## Reproductive risks of N_2_O

While describing the safety profile of N_2_O, our attention was drawn to reports dating back to the 1980s describing N_2_O-induced reproductive damage among healthcare personnel exposed to N_2_O. However, most of these professionals were also exposed to other general anaesthetics, like sevoflurane, and these also have reproductive risks (Schifilliti et al., 2011). Therefore, we focussed on N_2_O-related reproductive adverse effects in experimental animal studies and in observational studies in dental staff and midwives, that is, professionals that are generally not exposed to a mixture of anaesthetic gases. However, the results in early studies in dental assistants may be biased by exposure to amalgam, known to impair reproduction (Schuurs, 1999).

Rodents exposed to N_2_O showed a variety of developmental effects (increased resorptions, decreased foetal weight and malformations, like cleft palate, limb defects, gut herniation) (Dutch Health Council (DHC), 2000). More specifically, rodents exposed for 2–4 days to N_2_O (20–30% mixed with 20% O_2_ and 50–60% N_2_) showed decreased testis weight, impaired spermatogenesis and injury of the seminiferous tubules (Kripke et al., 1976), disrupted cycles after exposure of females and decreased fertility (Kugel et al., 1990). In another study, rodents exposed for 30 days to 0.5% N_2_O showed reduced litter size and smaller offspring in females mated with exposed males (Vieira et al., 1983). However, other studies failed to detect adverse effects of N_2_O on fertility. For a review on animal reproduction toxicity of N_2_O, see DHC (2000) and Menon et al. (2021).

Occupational exposure of dental assistants and midwifes to N_2_O appeared to be associated with congenital anomalies (Cohen et al., 1980), spontaneous abortion (Axelsson et al., 1996; Cohen et al., 1980; Nixon et al., 1979; Rowland et al., 1995) and reduced fertility rates (Ahlborg et al., 1996; Bennett, 2001; Rowland et al., 1992). For instance, in the study of Cohen et al. (1980) among 20,000 female dental assistants, exposure to N_2_O was associated with a 1.7–2.3 time increased risk for spontaneous abortion. A previous mail survey by Cohen et al. (1975) showed that spouses of male dentists (*n* = 7439), who were exposed to N_2_O in the year before conception, reported a 50% greater incidence of spontaneous abortion. A meta-analysis of 19 studies between 1971 and 1995 found a relative risk of spontaneous abortion after N_2_O exposure of 1.48 (95% confidence interval (CI): 1.41–1.58). In a selection of studies with the strongest design, the relative risk increased to 1.90 (95% CI: 1.72–2.09) (Boivin, 1997). Interestingly, foetal loss became only apparent when dental assistants were exposed to N_2_O for more than 3–5 working hours per week (Rowland et al., 1995), whereas reduced female fertility became evident in women with 5 or more hours of exposure per week (Rowland et al., 1992). The observed thresholds imply that spontaneous abortion and reduced fertility may occur only above a certain cumulative dose per week or above a certain minimum time of exposure (>3 h and >5 h per week, respectively).

The association between occupational N_2_O exposure of dentists and male infertility was not specifically studied. For a review on the effects of N_2_O exposure on reproductive outcomes in humans, see DHC (2000) and Olfert (2006).

While in the past, exposure levels up to 7500 ppm N_2_O (13,500 mg/m^3^) were measured in ambulances (Ancker et al., 1980) and dentist practices (Hillman et al., 1981), such high levels are no longer seen. Awareness about the reproductive harm of N_2_O has increased since occupational exposure limits (OELs) were set worldwide to protect healthcare personnel from unwanted exposure to ambient N_2_O (Sanders et al., 2008). The Dutch Health Council (DHC, 2000) has recommended to classify N_2_O as a substance that causes concern for human fertility (possible risk for impaired fertility) and development (possible risk of harm to the unborn child). Based on all available evidence for reproductive toxicity and adverse developmental outcomes in animals exposed to N_2_O, a health-based OEL of 20 mg/m^3^ (11 ppm) as 8 h time-weighted average was derived (Menon et al., 2021). In the United States, the NIOSH recommended a somewhat higher threshold limit value of 25 ppm or 46 mg/m^3^ (National Institute for Occupational Safety and Health (NIOSH), 2019).

## Biochemical basis of the reproductive risks of N_2_O

The pathogenesis of impaired reproduction by most volatile anaesthetics, like isoflurane and halothane, remains unclear and is likely to involve numerous membrane proteins and ion channels. In contrast, the mechanism of N_2_O-induced harm is well known as it inactivates vitamin B_12_ (cobalamin) (Sanders et al., 2008). Operating theatre nurses exposed to N_2_O (36–1502 mg/m^3^; exceeding the OEL) showed lower vitamin B_12_ plasma levels (437 vs 373 pM; *p* = 0.001) and higher homocysteine levels (11.2 vs 8.9 mM, *p* = 0.006) compared to medical personnel not exposed to N_2_O and these effects were N_2_O exposure level-dependent (Krajewski et al., 2007). It should, however, be noted that most of the subjects in the N_2_O-exposed group were simultaneously exposed to low levels of other volatile anaesthetics, for example, sevoflurane (0.2–21 mg/m^3^), but the exposure levels to these other anaesthetics were well below existing OELs, which makes confounding of the negative effect of N_2_O exposure on vitamin B_12_ levels by simultaneous exposure to the other anaesthetics unlikely.

**Figure 1.**
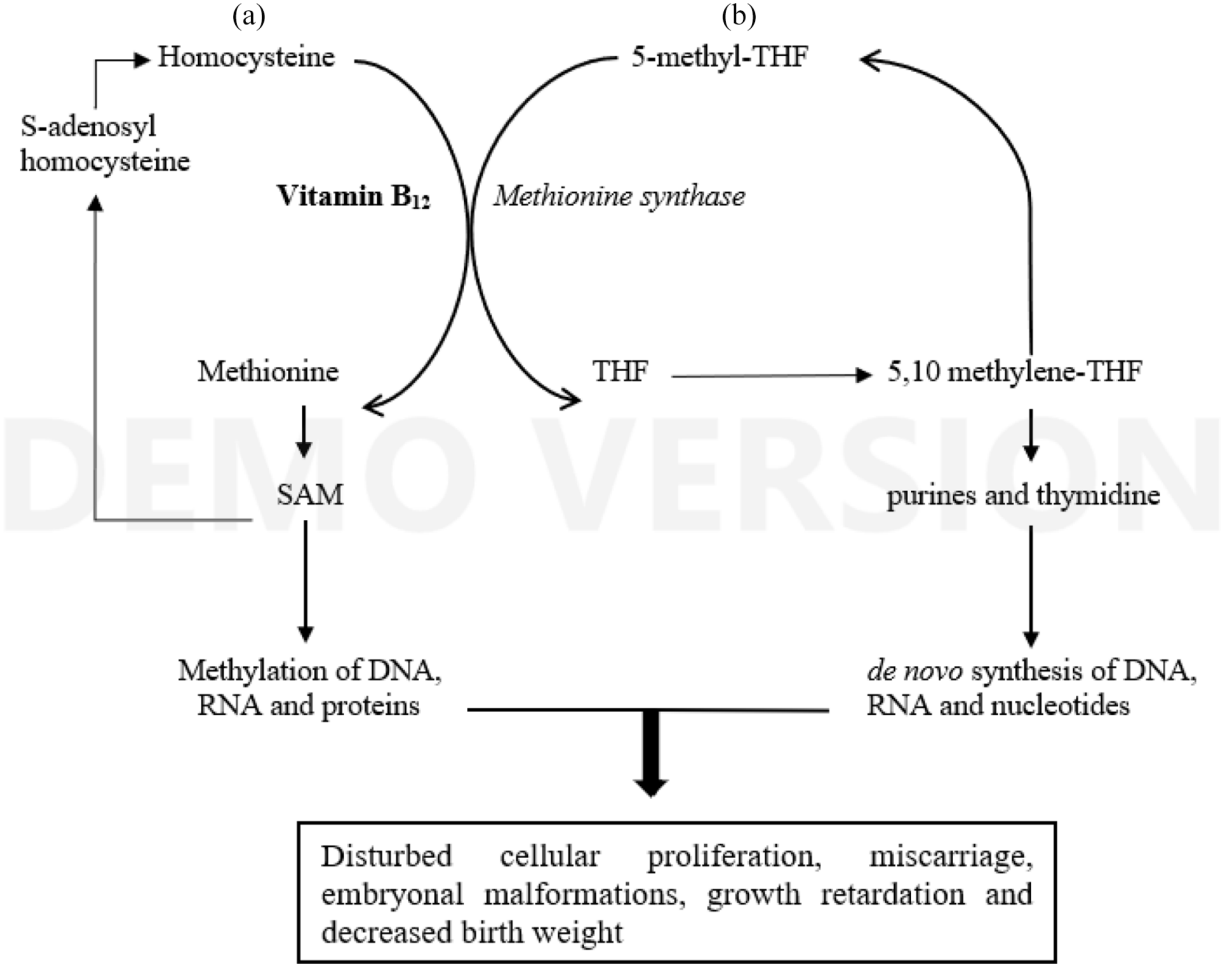
N_2_O inactivates vitamin B_12_, the essential cofactor of the enzyme methionine synthase: (a) the methylation cycle synthesizes S-adenosylmethionine (SAM), the universal methyl donor for transmethylation of DNA and other non-genomic methylation reactions; (b) the folate cycle serves, indirectly via tetrahydrofolate (THF) and 5,10 methylene-THF, the *de novo* synthesis of purines and thymidine, building blocks of nucleotides, DNA and RNA.

Vitamin B_12_ is an essential cofactor for methionine synthetase which plays a critical role in two key metabolic cycles in cell proliferation: the methylation cycle and the folate cycle (cf. Figure 1). In continuous recycling of homocysteine to methionine, catalysed by methionine synthetase, the methyl group of the substrate 5-methyl-tetrahydrofolate (5-methyl-THF; methyl folate) is transferred to homocysteine. The methionine formed is required for the biosynthesis of s-adenosylmethionine, an essential methyl donor in the synthesis of DNA, RNA, myelin and other proteins that contribute to gene expression and cell proliferation (Sanders et al., 2008; Vallejo and Zakowski, 2019). The folate cycle serves the *de novo* biosynthesis of thymidylate and purine, basic elements of DNA. Clearly, both cycles can be disrupted either by folate deficiency or inhibition (or deficiency) of vitamin B_12_, which results via DNA synthesis and impaired cellular proliferation and organogenesis, in genotoxicity and impaired reproductive functions, respectively. Indeed, in humans, exposure to N_2_O was found to be dose-dependently associated with DNA damage (Pasquini et al., 2001; Wrońska-Nofer et al., 2012). However, N_2_O-induced genotoxicity seems to be reversible due to DNA repair, since the increase of sister chromatid exchanges (marker of genotoxicity) reversed to normal values after a 2-month period of non-exposure (Eroglu et al., 2006). For a review on genotoxicity, see Schifilliti et al. (2011).

It is generally known that folate deficiency results in pregnancy-related complications and abnormal embryonic development with congenital defects, like neural tube defects (NTDs). However, low vitamin B_12_ status was also found to be associated with the risk of NTDs (Molloy et al., 2009), cleft lip/palate (Munger et al., 2021), early recurrent abortion (Reznikoff-Etiévant et al., 2002), preterm birth and low birth weight (Rogne et al., 2017). For review of vitamin B_12_ deficiency in pregnancy, see Van Sande et al. (2013). Effects of N_2_O on male reproductivity in rodents (Kripke et al., 1976) have already been described. In addition, it is relevant to mention the importance of vitamin B_12_ in male fertility. Vitamin B_12_ plays a substantial role in spermatogenesis, and hence in semen quality, because (a) vitamin B_12_ deficiency lowers testicular function leading to aplasia of sperms and impaired spermatogenesis in rodents (Kawata et al., 1992) and (b) human data showed that total vitamin B_12_ concentration in seminal plasma was significantly correlated with the sperm concentration (*r* = 0.42; *p* < 0.001) (Boxmeer et al., 2007). The positive effects of vitamin B_12_ treatment on sperm parameters have been reviewed by Banihani (2017).

Another route to reproduction disorders induced by N_2_O exposure is low vitamin B_12_-related megaloblastic anaemia (Amess et al., 1978) (cf. Figure 1). Severe anaemia (Hb < 6 g/dL) is associated with placenta previa, abruptio placenta, operative delivery and post-partum bleeding (Flessa, 1974) in the mother and with prematurity, spontaneous abortions, low birth weight and foetal deaths (Sifakis and Pharmakides, 2000).

## Relevance for recreational N_2_O users

It is widely accepted that the incidental recreational use of N_2_O elicits virtually no harm, although the genotoxic and the (related) reproductive risks have in this respect so far been neglected. Particularly, heavy use of hundreds of bulbs (one bulb contains 10 mL or 8000 mg of N_2_O in a pressurized liquid form) may be quite harmful.

Occupational exposure results from inhaling waste N_2_O that escapes from the patient’s mask to the work area. On average, one inhales over one 8 h day (480 min, at a frequency of 15 inhalations per minute and 0.5 L per inhalation) a volume of 3.6 m^3^ of air. Based on an OEL value of 20 mg/m^3^ as 8 h time-weighted average (Menon et al., 2021), the daily allowed inhaled dose of N_2_O for an employee is on average 3.6 × 20 mg N_2_O = 72 mg N_2_O. In recreational use, the inhalation of one bulb represents a dose of 8000 mg (8 g) of N_2_O, which is about 100 times the allowed daily occupational dose. Note that a recreational user fills one balloon with 8000 mg N_2_O (the filled balloon has a volume of 3.2 L) which is self-administered via 4–5 inhalations from the balloon. Although acute exposure to a high dose does not compare well with long-term occupational exposure to relatively low doses, this 100-fold difference in exposure is significant. Even if one realizes that an uncertainty factor of 50 was used to account for extrapolation from rats to humans (factor 10) and interindividual variability within the population (factor 5) to calculate the OEL (Menon et al., 2021), this 100-fold exposure is relevant within this comparison. Moreover, one should apply an addition uncertainty factor to account for the young age of many recreational users and their unfinished maturation. On the contrary, one-year occupational exposure to the maximally allowed value (OEL value) would lead to a cumulative dose of 16,000 mg (based on 220 full time working days per year) which equals the recreational dose of only two bulbs of 8000 mg. Moreover, medical treatment with N_2_O in dentistry or pregnancy can lead to relative high acute exposures, though an estimated volume of 15–25 L of N_2_O is routinely sufficient for clinical sedation which equals 5–8 balloons as used recreationally. However, such patients are at low risk, considering their low cumulative annual dose since they receive only 1–2 treatments per year. However, the threshold found with respect to reproductive harm, as reported by Rowland et al. (1992, 1995), seems irrelevant for heavy recreational users. First, because they are exposed to much higher levels of N_2_O. Second, it is evident that repeated inhalation of high levels of N_2_O from balloons for less than 3–5 h per week will not avoid the presumed reproductive harm.

It should be emphasized that this review provides only evidence for an association, but not for a causal relation, between N_2_O and impaired reproduction in humans. In addition, one may question how long-term, but intermittent exposure (occupational exposure) compares with repeated exposure to very high doses (compared to those occupational exposure; recreational exposure) with respect to reproductive harm. This is still a gap of knowledge to be further investigated. Furthermore, impaired reproduction in female recreational users of N_2_O is not yet reported. Finally, it seems that the risk for occasional users of N_2_O is very limited. In conclusion, the recreational user who frequently uses high doses of N_2_O should be aware of its potential genotoxic and reproductive risks, particularly when the exposed person is pregnant or intends to become pregnant. It is advocated to raise awareness of these risks among medical professionals through the medical curriculum and among recreational N_2_O users via public health campaigns.
